# Cumulative Human Impacts on Mediterranean and Black Sea Marine Ecosystems: Assessing Current Pressures and Opportunities

**DOI:** 10.1371/journal.pone.0079889

**Published:** 2013-12-04

**Authors:** Fiorenza Micheli, Benjamin S. Halpern, Shaun Walbridge, Saul Ciriaco, Francesco Ferretti, Simonetta Fraschetti, Rebecca Lewison, Leo Nykjaer, Andrew A. Rosenberg

**Affiliations:** 1 Hopkins Marine Station, Stanford University, Pacific Grove, California, United States of America; 2 National Center for Ecological Analysis and Synthesis, Santa Barbara, California, United States of America, and the Center for Marine Assessment and Planning, University of California Santa Barbara, Santa Barbara, California, United States of America; 3 ESRI, Redlands, California, United States of America; 4 Miramare Marine Protected Area, Trieste, Italy; 5 Laboratory of Marine Biology, Università del Salento, Lecce, Italy; 6 Coastal and Marine Institute Laboratory, San Diego State University, San Diego, California, United States of America; 7 European Commission, Joint Research Centre, Institute for Environment and Sustainability, Ispra, Italy; 8 Union of Concerned Scientists, Cambridge, Massachusetts, United States of America; Northwest Fisheries Science Center, NOAA Fisheries, United States of America

## Abstract

Management of marine ecosystems requires spatial information on current impacts. In several marine regions, including the Mediterranean and Black Sea, legal mandates and agreements to implement ecosystem-based management and spatial plans provide new opportunities to balance uses and protection of marine ecosystems. Analyses of the intensity and distribution of cumulative impacts of human activities directly connected to the ecological goals of these policy efforts are critically needed. Quantification and mapping of the cumulative impact of 22 drivers to 17 marine ecosystems reveals that 20% of the entire basin and 60–99% of the territorial waters of EU member states are heavily impacted, with high human impact occurring in all ecoregions and territorial waters. Less than 1% of these regions are relatively unaffected. This high impact results from multiple drivers, rather than one individual use or stressor, with climatic drivers (increasing temperature and UV, and acidification), demersal fishing, ship traffic, and, in coastal areas, pollution from land accounting for a majority of cumulative impacts. These results show that coordinated management of key areas and activities could significantly improve the condition of these marine ecosystems.

## Introduction

Ecosystem-based management (EBM) and Marine Spatial Planning (MSP) are widely being pursued as strategies to achieve the sustainable flow of marine ecosystem services [1], [2]. These comprehensive marine management frameworks are now mandated in some nations around the world [3], including Canada (Canada's Ocean Act of 1996), the USA (The National Ocean Policy of 2010), and Australia (http://www.gbrmpa.gov.au/zoning-permits-and-plans/zoning).

Member states of the European Union (EU) are also committed to adopting an ecosystem approach to marine management, including marine spatial planning. As mandated by the EU Marine Strategy Framework Directive (MSFD 2008), all European states should assess the environmental status of their territorial waters by July 2014, and develop strategies to achieve “good environmental status” by 2020 (GES, http://ec.europa.eu/environment/water/marine/es.htm). Marine spatial planning is also part of the EU Integrated Maritime Policy (IMP) of 2011 (http://ec.europa.eu/maritimeaffairs/policy/). At the regional scale, all the 21 Mediterranean nations have ratified the UNEP's Mediterranean Action Plan (MAP) to move Mediterranean marine management towards an ecosystem approach (ECAP), and to expand into non-EU Mediterranean waters the same conservation and management measures implemented in EU waters.

The Mediterranean and Black Sea ecosystems have been threatened by historical and current pressures e.g [4], [5], [6] which have led to major shifts in marine ecosystems and widespread conflict among marine users [7], [8], [9], [10]. Because of such intense pressure from multiple uses and stressors, the Mediterranean is characterized as a sea “under siege” [6], [11], and here, as in other intensely used ocean areas, an EBM approach has been recommended as a better management alternative to current sectoral management [1].

MAP ECAP, the EU MSFD and IMP provide an unprecedented opportunity to implement comprehensive and coordinated management of multiple uses and activities affecting the Mediterranean and Black Seas. These initiatives have no formal links, but a common timeline has recently been agreed upon [12]. It is expected that the implementation of the MAP ECAP will provide a platform for harmonization of national marine strategies of all Mediterranean countries (EU and non-EU) on a regional scale.

The development of basin-wide plans requires information on current impacts that can inform effective marine policy over the next years. Some relevant research has been conducted in this region. Coll et al. [6] have mapped cumulative impacts to key taxa and this assessment highlighted several areas of concern. However, a comprehensive analysis focused on whole ecosystems is lacking and quantification of the intensity and distribution of cumulative impacts in the whole Mediterranean and Black Sea that directly connects to ecosystem goals and priorities is critically needed.

We apply an approach developed to assess and map cumulative human impacts [13] that was previously applied to other marine regions, including the US EEZ e.g [14], [15], western Canada [16], and the North (http://harmony.dmu.dk/) and Baltic Seas [17]. Our goals are to: (1) quantify and map cumulative impacts to the entire Mediterranean and Black Sea to provide the data needed to guide and inform the development of effective marine policy; and (2) propose and apply a tool for assessing the environmental status of the territorial waters of EU member states. In particular, we identify the most and least impacted ecoregions and ecosystems within the Mediterranean and Black Sea, the top threats affecting EU territorial waters and the entire basin, and the areas that represent top priorities for EBM and conservation efforts. These analyses are aimed at supporting coordinated and comprehensive actions across the basins, ensuring GES consider all impacts and are relevant at both the national and the regional scales.

## Methods

We used a cumulative impact model that follows a 4-step process [13]. We first assembled spatial datasets for *n* = 22 anthropogenic drivers (*D_i_*) and *m* = 17 ecosystems (*E_j_*) (Table S1 and Figs. S2–S11 in File SI). All but the climatic drivers have direct correspondence with the MSFD's good environmental status (GES) descriptors (Table S2 in File SI). Second, we log[X+1]-transformed and rescaled between 0–1 each driver layer to put them on a single, unitless scale that allows direct comparison, and converted ecosystem data into 1 km^2^ presence/absence layers. Third, we calculated cumulative impact scores (*I_C_*) for each 1 km^2^ pixel as 
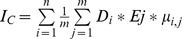
, where *μ_i,j_* is the impact weight for anthropogenic driver *i* and ecosystem *j*, and *1/m* produces an average impact score across ecosystems [13]. Impact weights were estimated using expert judgment to quantify vulnerability of ecosystems to human drivers of ecological change [18]. Although these weights are not specific to the Mediterranean, regional experts were included in those analyses, and weights have proven fairly consistent for other regional assessments [19], [20]. The use of expert judgment instead of direct empirical assessments to calculate impact weights greatly increases uncertainty of our impact scores. Empirical quantification of the ecological impacts of a suite of drivers is currently unavailable and filling this gap is a critical need within the Mediterranean and other regions [10], [18]. Despite these limitations, there is a long history in the decision sciences of assessing how to set priorities (e.g., rank threats) by using the best available scientific judgments when data are scarce and uncertainty exists. Teck et al. [19] critically examined uncertainties associated with expert judgment and showed the robustness of our approach to eliciting expert opinions for informing cumulative impact assessments for the California Current. Based on these results, we used the same approach and vulnerability weights here. Regardless, it is critical that uncertainties associated with a lack of empirical data on ecosystem vulnerability are communicated clearly, especially when integrating cumulative impact mapping into decision making [21].

Cumulative impact to individual ecosystems (*I_E_*) was calculated as, 
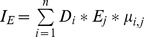
 and impact of individual drivers across all ecosystem types (*I_D_*) was calculated as 
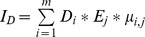
. Finally, we used the same thresholds used in Halpern et al. [13] to designate ecologically meaningful categories of the cumulative impact scores, i.e. ecosystems that are subject to: very high (*Ic*>15.52); high (12–15.52); medium high (8.47–12); medium (4.95–8.47); low (1.4–4.95); and very low impact (<1.4). These thresholds were based on empirical data on the condition of ecosystems containing coral reefs, seagrass beds, mangroves and surrounding matrix of soft bottom habitats [13]. In Halpern et al. [13], cumulative impact scores were translated into estimates of ocean condition by using linear regression to compare estimates of the current condition of 16 regions containing coral reefs from around the world [23] to the average cumulative impact score for all cells containing coral reefs in those regions. The statistically significant linear regression equation was then used to translate impact scores into categories of ocean condition. We used these bins of cumulative impact scores for describing the condition of Mediterranean marine ecosystems and for color-coding all figures. Categorization of cumulative impact scores in very low to very high impact classes does not imply a value judgment, but is aimed instead at enabling analyses and visual display of results.

We used these thresholds for categorizing level of threat, even though they were derived in different regions and ecosystem types, because a similar in-situ verification of the Mediterranean ecosystem condition currently does not exist. The sensitivity of our results to key steps in this process and further details on the validation method were analyzed in previous articles and results were shown to be robust to variation in weights and thresholds [13], [19]. However, the assumption that thresholds derived from tropical coastal ecosystems apply to Mediterranean habitats and to offshore and deep ecosystems was not directly validated. Empirical ground-truthing of the relationship between cumulative impact scores and ecosystem condition remains a top priority within the Mediterranean and worldwide [13], [14], [21].

We first calculated and mapped *Ic* over the entire Mediterranean and Black Sea basins. We also calculated impact scores separately for each ecoregion, sensu [22] (see Table 1), and for each of four categories of drivers: climatic (temperature and UV increase, and acidification), land-based (nutrient input, organic pollution, urban runoff, risk of hypoxia and coastal population density), sea-based (commercial shipping, invasive species, oil spills and oil rigs), and fishing (all fishing gears and types) (Table S1 in File SI).

**Table 1:** Average, maximum, SD and CV of the cumulative impact scores within the seven Mediterranean ecoregions and the Black Sea.

Ecoregion	avg. *Ic*	max *Ic*	SD	CV
Alboran Sea	9.1	20.2	2.0	22.4
Levantine Sea	8.9	19.2	2.5	27.8
Aegean Sea	8.6	18.5	2.6	29.9
Adriatic Sea	8.4	19.0	3.4	40.9
Tunisian Plateau/Gulf of Sidra	8.3	21.0	2.6	31.1
Ionian Sea	8.3	24.5	1.7	20.4
Western Mediterranean	7.7	22.4	1.9	24.7
Black Sea	6.1	16.4	2.2	35.9

Secondly, we calculated and mapped *Ic* only for the territorial waters of the EU member states, (up to 12 nm from the coastline) where more data are available. Analyses were conducted at two different spatial scales – only for EU member states and for the entire Mediterranean and Black Sea – to account for the spatial scale of available data layers. All 22 drivers were used in the EU analysis but only 18 in the Mediterranean and Black Sea-wide analysis because data on coastal erosion, renourishment, engineering, and urbanization trends were available only for EU member states (Table S1 in File SI). Per-pixel *Ic* scores ranged between 0–24.5 in the Mediterranean-wide analysis, and 0.2–97.3 for the EU analysis. Sources and methods used to develop each data layer are detailed in the SOM (Text S1 in File SI).

## Results

### Mediterranean and Black Sea

**Figure 1:**
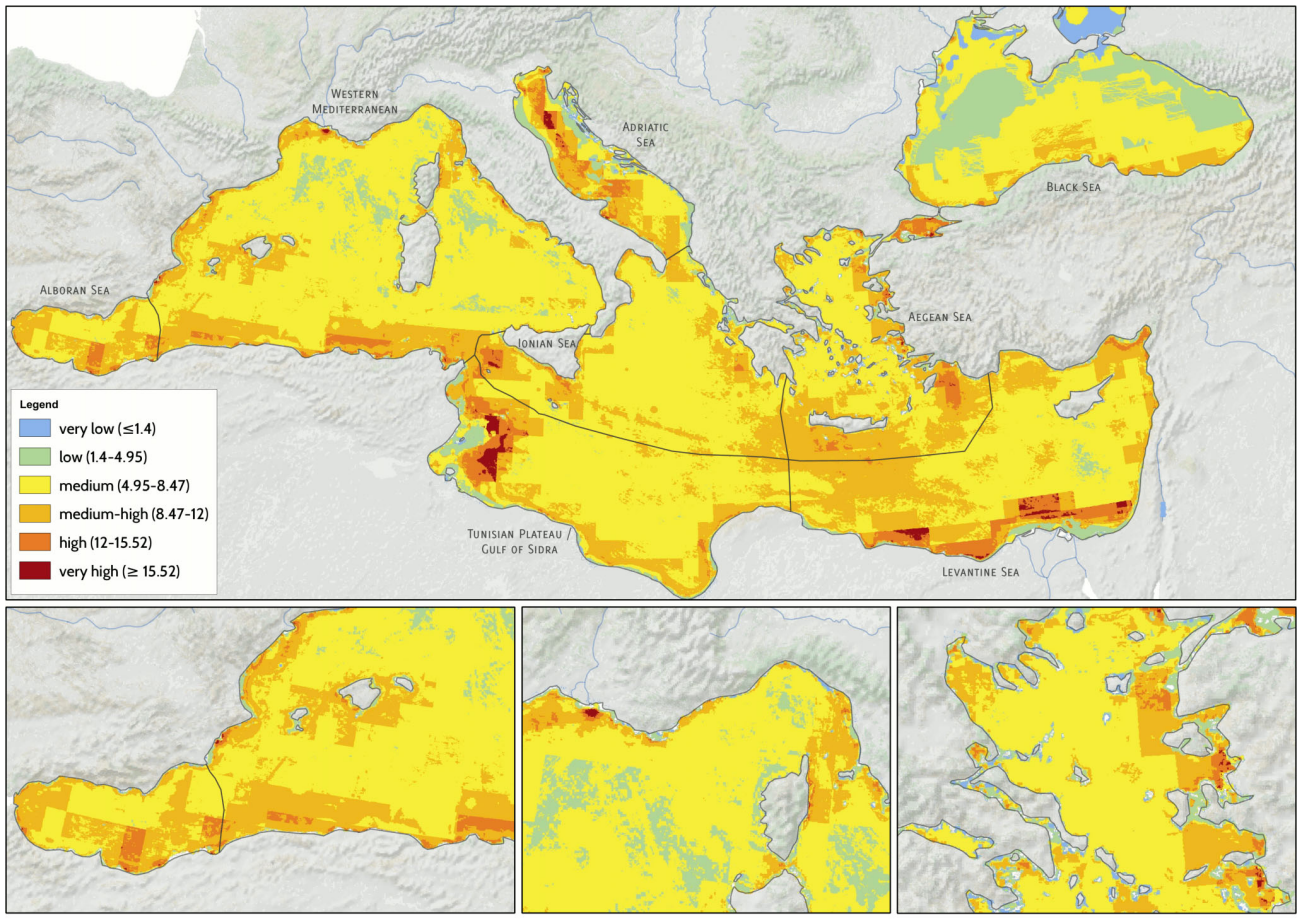
Spatial distribution of cumulative impacts to marine ecosystems of the Mediterranean and Black Sea. Inserts at the bottom show larger views of the Alboran (left), Northern Tyrrhenian (center), and Aegean Sea (right). Colors correspond to the different impact categories listed in the legend.

The map of cumulative human impacts highlights the widespread distribution of drivers, and resulting impacts, throughout the Mediterranean and Black Sea (Fig. 1). Regions of medium-high to very high impact (20.5% of the total area) are found within the Alboran Sea, the Gulf of Lyons, the Sicily Channel and Tunisian Plateau, the Adriatic Sea, off the coasts of Egypt and Israel, along the coasts of Turkey, and within the Marmara and Black Sea (Fig. 1). Areas of very-low to low impact account for a total 13.6% of the total surface area, and are present within the central Tyrrhenian Sea, parts of the northern and central Adriatic Sea, the southern Levantine Sea, and the eastern and western sides of the Black Sea (Fig. 1). A majority (65.9%) of the Mediterranean and Black Sea are subject to medium cumulative impact.

When analyzed by ecoregion, the Alboran and Levantine ecoregions have the highest average cumulative impact, the western Mediterranean and Black Sea the lowest (Table 1), although areas of high impact exist even within these ecoregions (Fig. 1). In fact, the greatest per-pixel *Ic* scores were seen within the western Mediterranean and in the Ionian Sea, and the Adriatic and Black Seas exhibited the greatest variability in impact scores (Table 1), comprising areas of both very high and low impact (Fig. 1).

Pelagic and benthic offshore ecosystems have the greatest average cumulative impact (*I_E_*; Table S3 in File SI). However, this is partly driven by their large extent: the maximum pixel-level values of the *I_E_* scores, indicative of locally high impacts, are observed in intertidal habitats (particularly salt marshes, suspension-feeding reefs, rocky shores and mud flats), and in nearshore sublittoral and continental shelf hard bottoms that are affected by both sea-based and land-based drivers (Table S3 in File SI).

### EU member states

Quantification of the percent of national waters of EU member states in different impact categories (Table 2) reveals that a majority (60–99%) of waters within 12 nm of the coastline are subject to medium-high to very high impact. 0–20% of national waters are subject to low or very low impact, and this percent is less than 10% for a majority of nations (Table 2). Average *Ic* (averaged by nation) range from 6.2–15.3, corresponding to medium to high impact (Table S4 in File SI), and show no correlation with the area of national waters (*R^2^* = 0.0005, NS).

**Table 2:** Percent of national territorial waters of Mediterranean and Black Sea EU member states within different impact categories: very high impact (*I_c_*>15.52); high impact (12–15.52); medium-high impact (8.47–12); medium impact (4.95–8.47); low impact (1.4–4.95); and very low impact (<1.4).

Country	area (km^2^)	very low	low	med	med-high	high	very high
Slovenia	266.2	0.0	10.2	22.7	0.0	0.8	66.3
Cyprus	95,833.6	1.8	4.4	0.7	12.2	63.3	17.6
France	480,103.7	0.4	4.1	26.2	26.7	27.6	14.9
Italy	700,184.6	0.0	6.3	14.0	44.6	21.4	13.7
Spain	744,352.6	0.0	6.6	7.2	40.0	33.9	12.2
Bulgaria	48.050.1	5.3	14.9	15.7	33.6	22.5	8.0
Greece	615,025.4	0.8	9.0	9.7	51.3	21.3	7.9
Monaco	390.6	0.0	0.0	0.8	60.2	32.8	6.3
Malta	68,240.6	0.9	2.9	37.5	34.8	19.0	4.8
Romania	41,509.7	1.4	16.5	20.5	47.5	12.8	1.2

**Figure 2:**
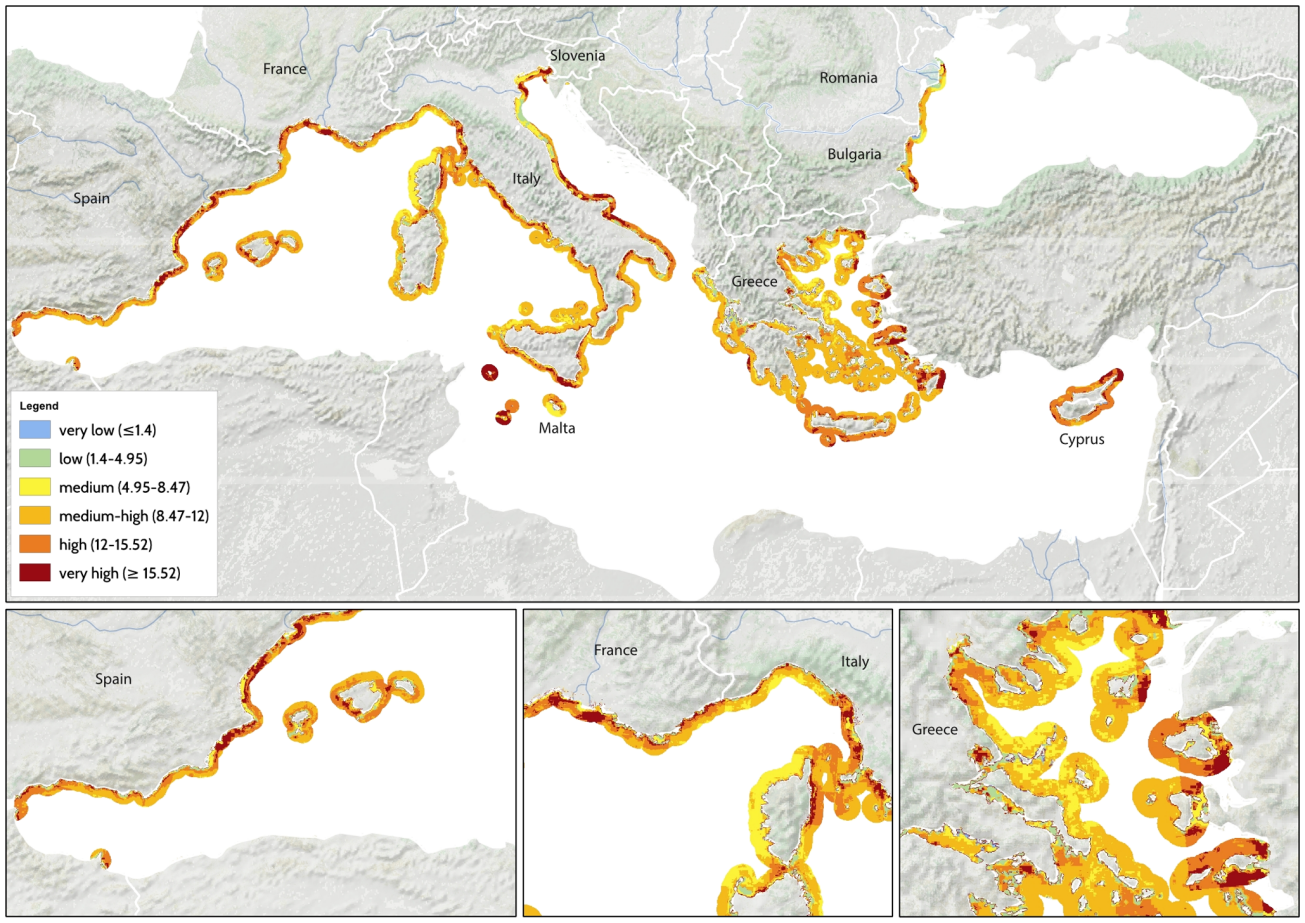
Spatial distribution of cumulative impacts to the territorial waters of EU member states. Inserts at the bottom show larger views of the Alboran (left), Northern Tyrrhenian (center), and Aegean Seas (right). Colors correspond to the different impact categories listed in the legend. Territorial waters extend 12 nm from the coastline.

Cumulative impact maps show high spatial variation in impact scores, with heavily impacted systems (in red) found within the waters of all nations, and small areas with relatively low impact (in blue or green) along the coasts of most nations (Fig. 2). The addition of four coastal drivers to this analysis (coastal erosion, renourishment, engineering, and urbanization trends) results in greater *Ic* scores in most locations compared to the Mediterranean-wide map (Figs. 1–2).

### Relative contribution and spatial distribution of drivers

**Figure 3:**
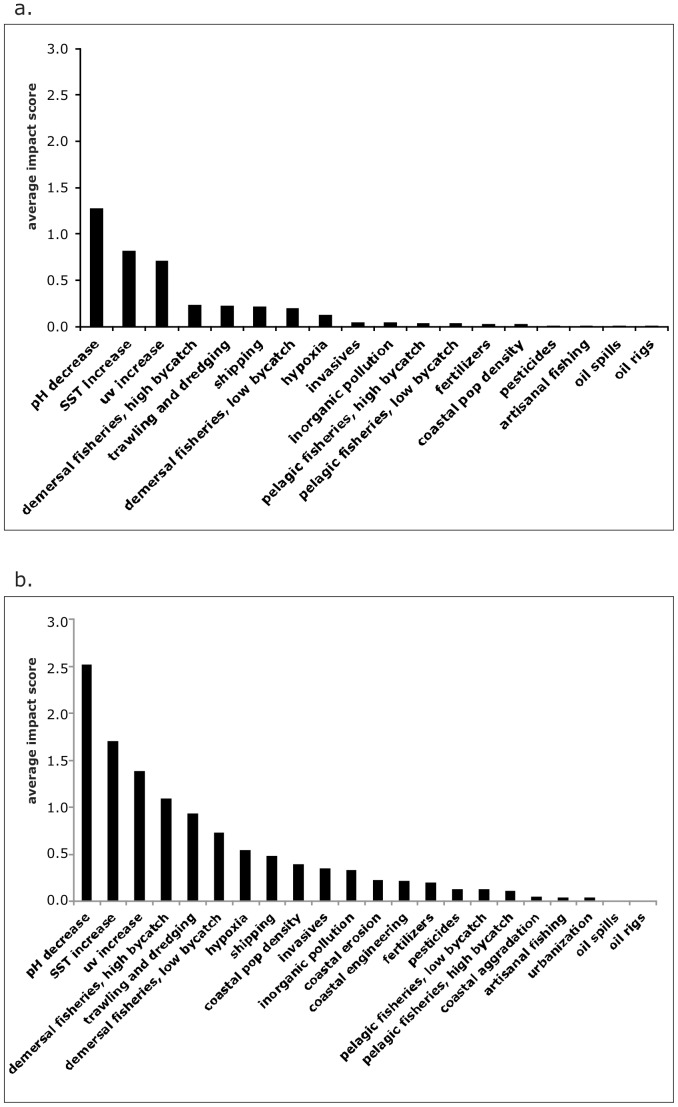
Average Impact scores of drivers. Average impact scores for each driver are reported within (a) the entire Mediterranean and Black Sea, and (b) the territorial waters of EU member states.

Drivers associated with climate change (SST and UV increase, and acidification), demersal fishing, and shipping result in the greatest average impact on Mediterranean and Black Sea ecosystems (Fig. 3a). These drivers, along with coastal hypoxia, were found to exert the greatest impact within territorial waters of the EU nations (Fig. 3b), followed by coastal population density, invasive species, land-based pollution (inorganic pollution, pesticide and fertilizer runoff) and modification of the coastline (through coastal erosion and engineering). The lowest estimated impacts, both within EU waters and for the whole basin, are associated with oil spills and rigs (Fig. 3).

**Figure 4:**
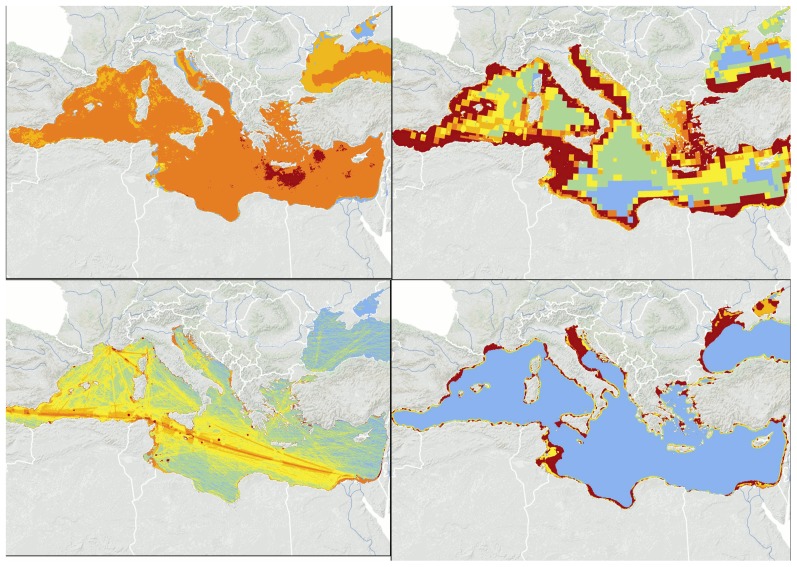
Spatial distribution of cumulative impact of driver categories. Driver categories are: climate (i.e. the combined cumulative impact of temperature and UV increase, and acidification; top left), fishing (all fishing types combined; top right), sea-based drivers (commercial shipping, invasive species, oil spills and oil rigs; bottom left) and land-based drivers (nutrient input, organic pollution, urban runoff, risk of hypoxia and coastal population density; bottom right). Color scales correspond to highest (red) to lowest (blue) cumulative impact, within each panel. Different scales were used in each panel to better highlight spatial patterns for each driver category.

Driver categories differ in their distribution across the Mediterranean and Black Sea (Fig. 4; Table S1 in File SI). Climatic drivers are broadly distributed but have the greatest impact scores in the eastern Mediterranean. Fishing affects all coastal areas, as well as most of the Sicily Channel and the Alboran, Adriatic, and Aegean Sea. Sea-based activities result in the highest scores in the western and southern regions, and land-based activities broadly affect coastal areas, as well as large portions of the Adriatic and Black Sea.

**Figure 5:**
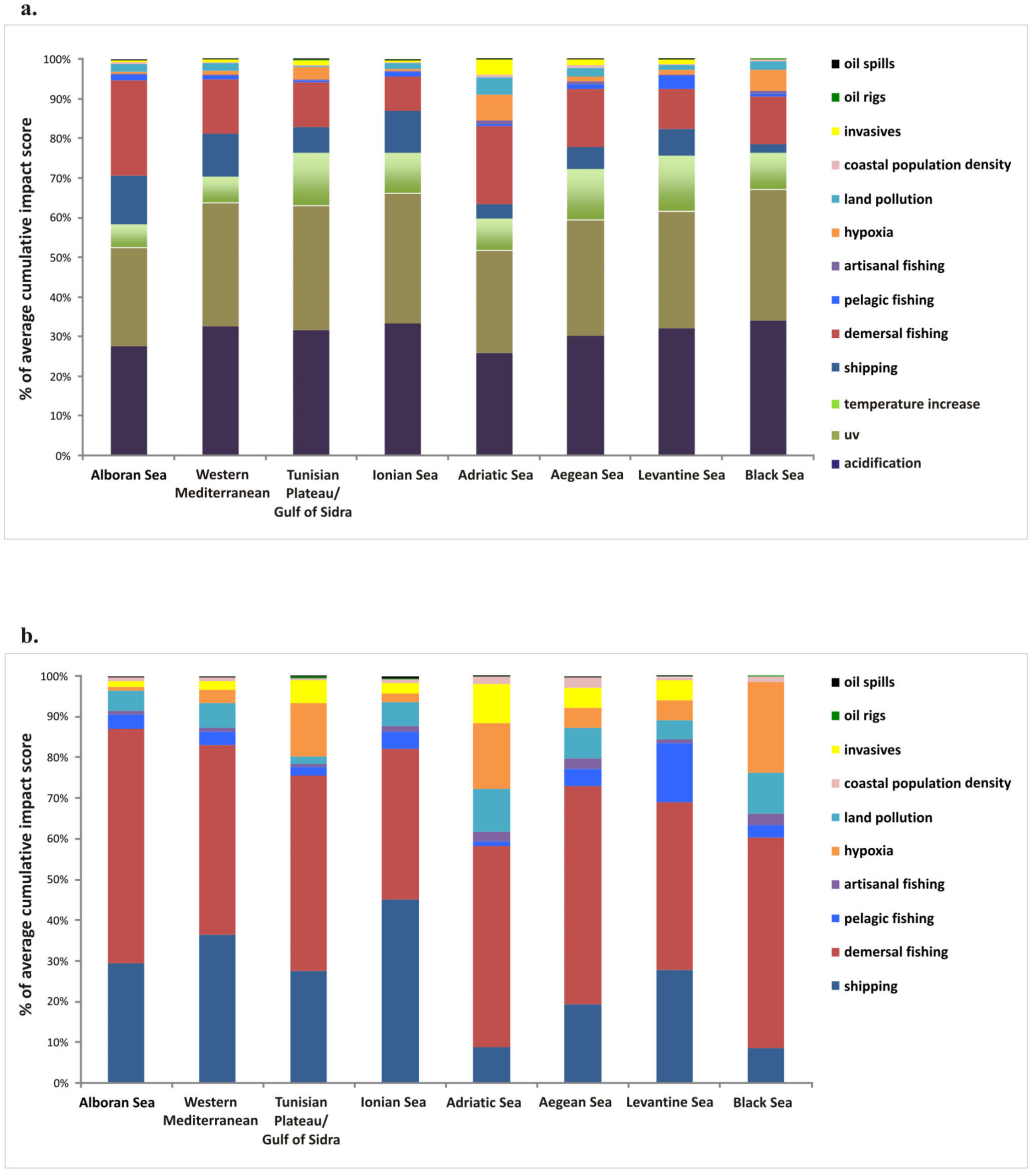
Percent contribution of different drivers to the average cumulative impact score of each ecoregion. Percent contributions are reported with (a) and without (b) climatic drivers. Some drivers (different demersal and pelagic fisheries, and different types of pollution from land) are aggregated to show relative contributions more clearly.

All ecoregions are affected by multiple drivers, but the relative importance of different drivers varies among ecoregions (Fig. 5). Climatic drivers have the greatest contribution to the average cumulative impact score of all ecoregions, though this contribution is lower for the Alboran and Adriatic Seas (Fig. 5a). When climatic drivers are not included (Fig. 5b), demersal fishing, hypoxia and pollution from land-based activities are major contributors to high cumulative impacts to the Adriatic and Black Sea, and demersal fishing and shipping are major contributors in the other ecoregions.

## Discussion

Our analysis highlights that 20% of the entire Mediterranean and Black Sea and 60–99% of the territorial waters of EU member states are subject to high impact, while less than 20% has low impact and very few areas, less than 1%, remain relatively unaffected by human activities. Cumulative impact varies greatly across and within ecoregions and countries. Highly impacted areas are found in the Alboran Sea, the Gulf of Lyons, the Sicily Channel and Tunisian Plateau, the Adriatic Sea, off the coasts of Egypt and Israel, along the coasts of Turkey, and within the Marmara and Black Sea (Figs. 1 and 2). Contemporarily, we highlight areas characterized by low cumulative human impacts off Croatia, Albania, Italy, Tunisia and Egypt, in offshore areas of the central Tyrrhenian and Black Sea, and in several small areas along the coasts of most countries (Figs. 1 and 2). These areas represent important opportunities for conservation aimed at preventing future degradation.

Some of the highly impacted areas we identified in this analysis coincide with the areas of conservation concern identified by Coll et al. [6], including portions of the Northern Adriatic Sea, the Sicily Channel, the inner Ionian Sea, the Aegean Sea, and the Gulf of Lyons. These areas emerge as clear priorities for future management action and protection. However, our analyses highlight additional highly impacted areas where multiple drivers overlap with vulnerable habitats, e.g. in the central Adriatic Sea, the Alboran Sea, the Tunisian Plateau, and in the southern Levantine Sea. These areas may also represent high priorities for management and conservation action. By using habitats as proxies for biodiversity instead of species data, our approach allows for the identification of areas of conservation concern in relatively data poor regions, such as the southern and eastern Mediterranean Sea, where species data are still scarce [6]. Taken together, results from Coll et al.'s [6] and our study show how multiple, complementary approaches are needed to assess cumulative impact and to direct research and management efforts to the areas that most urgently need them.

High cumulative impact scores were always associated with multiple drivers, supporting the need for coordinated, comprehensive plans addressing all drivers of ecosystem change across the Mediterranean and Black Sea. However, our results also highlight opportunities for a major reduction of cumulative impact by prioritizing a subset of the drivers for policy action. Demersal fisheries, ship traffic, and, in some coastal areas, fertilizer run-off and resulting hypoxia are major contributors to the cumulative impacts we have analyzed. While it is well known that these activities are important pressures on ecosystems worldwide, this analysis shows that reducing the effects of trawling, ship traffic and nutrient loading from some land-based activities could lead to large reduction in cumulative impact, relative to addressing other drivers. Furthermore, different management policies will be most effective in different regions. For example, in the Sicily Channel and Alboran Sea, a better environmental status could be achieved by focusing on spatial plans for fisheries and commercial shipping, and in the northern Adriatic Sea and the Black Sea on fisheries and pollution from land. In all regions, high spatial variability in the distribution of human pressures and vulnerable ecosystems provide opportunities to reduce cumulative impact through spatial planning of current and emerging activities.

In accord with previous global and regional impacts research [13]–[15] climatic drivers (temperature and UV increase, and ocean acidification) were found to cause the largest potential impacts. While climatic drivers cannot be removed through local management action, their spatial distribution highlights the areas where climate mitigation and adaptation is most critical, e.g., in the eastern Mediterranean Sea.

The estimated high impacts of major climatic and human drivers are influenced by both their widespread distribution (particularly climatic stressors), and the high vulnerability of multiple ecosystem types to these pressures e.g., [18]. In contrast, oil spills and rigs had the lowest estimated impacts, both within EU waters and for the whole basin. These highly harmful marine stressors showed in the Mediterranean a smaller effect because of their limited current spatial extent overlapping with habitats relatively resistant to oil-associated threats. Episodic and unpredictable, oil spills do not represent a chronic stressor to these ocean regions although a large-scale spill would clearly be catastrophic and damaging for these ecosystems.

Cumulative impact analyses and mapping can help inform policy reform and management plans by highlighting top priorities and possible opportunities for initiating EBM and MSP, and by providing imagery to facilitate communication of issues and opportunities to policy makers, environmental managers, conservationists, businesses and the public. However, several issues require attention when interpreting results from our analyses. First, major sources of uncertainty remain to be addressed in future empirical studies and modeling efforts [13], [16], [21]. Empirical information on how ecosystems change in response to different combinations and intensities of drivers is still scarce [10]. A better understanding of if or where ecosystems experience non-linear responses to cumulative impact and thresholds of resistance would be particularly valuable for setting management targets or limits. At present, non-linearities are difficult to anticipate and interpret and adaptive management responses that are robust to unexpected outcomes are needed. Data on the consequences of non-linear behaviors have never been included in Mediterranean analyses and rarely in extra Mediterranean areas [24]–[26]. Direct empirical assessments of the vulnerability of different ecosystems, in addition to expert judgments, and of the relationship between cumulative impact scores and ecosystem conditions are critically needed. Second, the quality of available data is widely variable, with a great need for improved spatial information on the distribution of different ecosystems, direct measures of fishing effort, and the distribution and effects of important drivers such as marine litter, bycatch, and aquaculture. The effects of data gaps can be seen by comparing the Mediterranean wide and EU analyses (Figs. 1–2): addition of only four data layers in the latter analysis results in greater cumulative impact to coastal areas, indicating that impact is underestimated in the broader analysis. As additional spatial data e.g., [27] become available, they could be incorporated in new iterations of these analyses. It will be especially important to perform even more detailed analyses using all locally available information in the top priority areas highlighted here. Third, our use of fisheries catch data [13] (Fig. S1 in File SI) instead of effort may lead to overestimation of impact in highly productive regions (e.g., the Alboran Sea) and underestimation in regions, such as parts of the Adriatic Sea, subject to intense historical fishing pressure and thus currently depleted [4], [28]. Improved access to effort data is a key priority: despite the Mediterranean Sea is a public trust resource and fishery-related data are collected with public funding, much of these data remain confidential. When spatially-explicit effort data become available they can and should be incorporated into future assessments. Fourth, our approach results in a static view of impact. Analyses of trajectories of change [9], [28] and tools such as InVEST (http://www.naturalcapitalproject.org) that simulate consequences of different management actions into the future would improve management plans and MSP at local and regional scales. Our analysis serves as a baseline against which future actions can be measured. Finally, and most critically, institutions and social processes play a key role in advancing marine management that no amount of data and technical sophistication can replace. Securing social and political acceptance of conservation and management initiatives and establishing effective processes for their implementation is critical [29]. Direct engagement of scientists and conservation practicioners in the planning process, analysis of social and economic costs and benefits of different management options, and involvement of diverse stakeholders will be essential to the successful implementation of marine spatial plans. Advancing marine conservation and management will require these fundamental participatory processes [29].

Achieving the environmental goals that nations have committed to, within the Mediterranean region and worldwide, will require ongoing and comprehensive monitoring of impacts in conjunction with new policies that balance biodiversity protection with human uses. Within the EU the European Commission has stimulated the development of MSP among the EU member states, and the debate is ongoing on how to implement the key principles of MSP [30], [31]. In parallel to these efforts, MAP is striving to expand the development of MSP into non-EU Mediterranean waters.

The impact assessment approach described here allows for a transparent, repeatable and updatable synthesis and integration of disparate information, facilitating communication and discussion of policy options and alternatives at different spatial scales. Cumulative impact assessment highlights that coordinated management of key areas and activities could significantly enhance the environmental condition of intensely used marine regions. Therefore cumulative impact assessment could be considered as one of the valuable tools for achieving the objectives of the EU maritime policy and MAP.

## Supporting Information

File SI **Detailed description of methods and spatial data layers, additional summary statistics of cumulative impact scores, and comparison of individual impact data layers from the global cumulative impact analysis** [**13**] **with the new layers included in this analysis.** **Text S1.** Methods; **Table S1.** List and characteristics of spatial datalayers; **Table S2.** Correspondence between descriptors of Good Environmental Status and the data layers used in this analysis; **Table S3.** Summary statistics of cumulative impact score for individual ecosystems; **Table S4.** Summary statistics of cumulative impact scores within territorial waters of Mediterranean and Black Sea EU member states; **Figure S1.** Correlation between fishing effort and catch for pelagic longlines; **Figure S2.** Modeled distribution of marine invasive species from Halpern et al. (2008); **Figure S3.** New data layer on the distribution of harmful invasive species in Mediterranean coastal waters; **Figure S4.** Modeled pollution at sea layer (Halpern et al. 2008); **Figure S5.** Occurrence and magnitude of accidents resulting in oil release; **Figure S6.** Frequency of positive temperature anomalies (Halpern et al. 2008); **Figure S7.** Annual average rate of increase in SST; **Figure S8.** Risk of hypoxia; **Figure S9.** Coastal erosion and aggradation along the coastlines of Mediterranean EU countries; **Figure S10.** Coastal armoring along the coastlines of Mediterranean EU countries; **Figure S11.** Urbanization trends within Mediterranean EU countries.
(PDF)
