# Supplementary material for: Cumulative Human Impacts on Mediterranean and Black Sea Marine Ecosystems: Assessing Current Pressures and Opportunities
Source: PLoS One. 2013 Dec 4;8(12):e79889. doi: 10.1371/journal.pone.0079889 (PMC3850916; doi:10.1371/journal.pone.0079889)
Supplement: File SI — Detailed description of methods and spatial data layers, additional summary statistics of cumulative impact scores, and comparison of individual impact data layers from the global cumulative impact analysis [13] with the new layers included in this analysis. Text S1. Methods; Table S1. List and characteristics of spatial datalayers; Table S2. Correspondence between descriptors of Good Environmental Status and the data layers used in this analysis; Table S3. Summary statistics of cumulative impact score for individual ecosystems; Table S4. Summary statistics of cumulative impact scores within territorial waters of Mediterranean and Black Sea EU member states; Figure S1. Correlation between fishing effort and catch for pelagic longlines; Figure S2. Modeled distribution of marine invasive species from Halpern et al. (2008); Figure S3. New data layer on the distribution of harmful invasive species in Mediterranean coastal waters; Figure S4. Modeled pollution at sea layer (Halpern et al. 2008); Figure S5. Occurrence and magnitude of accidents resulting in oil release; Figure S6. Frequency of positive temperature anomalies (Halpern et al. 2008); Figure S7. Annual average rate of increase in SST; Figure S8. Risk of hypoxia; Figure S9. Coastal erosion and aggradation along the coastlines of Mediterranean EU countries; Figure S10. Coastal armoring along the coastlines of Mediterranean EU countries; Figure S11. Urbanization trends within Mediterranean EU countries. (PDF) [file pone.0079889.s001.pdf]

Supplementary Online Materials

Methods

General Approach

We used a cumulative impact model that follows a 4-step process (Halpern et al. 2008; Selkoe et al. 2009). We first assembled the spatial data for each anthropogenic driver (D_i) and each ecosystem (E_j). Second, we log[X+1]-transformed and rescaled between 0-1 each driver layer to put them on a single, unitless scale that allows direct comparison, and converted ecosystem data into 1 km^2 presence/absence layers. Third, for each 1 km^2 cell of ocean we multiplied each driver layer with each ecosystem layer to create driver-by-ecosystem combinations, and then multiplied these combinations by the appropriate weighting variable (u_{ij}). These weighting variables come from an expert survey that assessed the vulnerability of each ecosystem to each driver on the basis of 5 ecological traits (Halpern et al. 2007). The weighting values represent the relative impact of an anthropogenic driver on an ecosystem within a given cell when both exist in that cell, and do not represent the relative global impact of a driver or the overall status of an ecosystem. The sum of these weighted driver-by-ecosystem combinations then represents the relative cumulative impact of human activities on all ecosystems in a particular 1 km^2 cell. Finally, we used the same thresholds used in the global analysis to designate ecologically meaningful categories of the relative cumulative impact scores. In the global analysis, these were based on empirical data on the condition of coral reef ecosystems (Halpern et al. 2008). The sensitivity of our results to key steps in this process and further details on the groundtruthing method was analysed in previous articles (Halpern et al. 2008; 2009).

In the present analysis we replaced some of the datalayers and included additional data to better reflect the specific pressures and ecosystems of the Mediterranean basin. A total of 22 spatial datasets of drivers (Table S1) and 17 ecosystem types (all the ecosystem layers used in Halpern et

al. 2008 except for those that do not exist in the Mediterranean Sea, i.e. the coral, mangrove and ice ecosystems) were assembled and used in the analyses and maps.

Some of the dalalayers measure impacts that are to some extent overlapping. In particular, nutrient input increases the risk of hypoxia, and these datalayers could be viewed as measuring the same impact on coastal marine ecosystem. However, each measures also distinct impacts. While nutrient runoff is a major contributor to coastal hypoxia, other factors also determine the occurrence of hypoxia (e.g. hydrodynamics, primary productivity and water temperature, all affecting the formation and degradation of organic matter). These factors are included in the OxyRisk model that generated our risk of hypoxia datalayer (Djavidnia et al. 2005). Therefore, coastal areas receiving high nutrient loadings do not necessarily develop hypoxic waters, depending on the physical and biological setting. However, high nutrient loadings can have other impacts, beyond hypoxia, including altering primary productivity, shifting the composition of planktonic and macroalgal assemblages, and affecting benthic invertebrate assemblages and associated food webs. Thus, we included both datalayers (risk of hypoxia and nutrient input) in our analysis because they capture complementary impacts of increased nutrient availability. Similarly, coastal population density and urbanization trends result in increased nutrient loading, but also in a suite of direct and indirect ecological impacts, through direct disturbance of people visitation and use of the shores (e.g., trampling, noise, collecting, littering) and habitat modification (conversion of natural habitat to hard structures, removal of coastal vegetation). Moreover coastal engineering results in the construction of structures that modify coastal circulation and sediment dynamics, in addition to replacing natural habitats. So in this case as well, we included all of these datalayers because they capture different impacts of coastal ecosystems, in addition to overlapping ones.

Maps of individual data layers and of the full cumulative impact model can be viewed at the public website:

http://globalmarine.nceas.ucsb.edu/mediterranean/

We conducted both a Mediterranean-wide analysis (displayed on the website above) and a more detailed analysis of cumulative impacts to the territorial waters (within 12 nm from the coastline) of Mediterranean and Black Sea countries that belong to the European Union (EU). This analysis used all of the data layers used in the first analysis and four additional layers (Table S1) that were available only for the EU member states.

A detailed description of data layers obtained from the previous global analysis can be found in the SOM of Halpern et al. 2008. We attempted to obtain or model measures of fishing efforts from commercial fleets and artisanal fisheries, but ended up using commercial fisheries catch statistics instead because actual effort data are available only for pelagic longline fisheries (data provided by one of the authors, Rebecca Lewison, UCSD, USA) but not for other fisheries. For pelagic longlines, we compared effort and catch, after aggregating datasets on the same coarse scale (5 degree cells) at which the effort data are reported (Fig. S1). Although visual inspection reveals a weak trend towards a positive correlation between effort and catch, this relationship is not statistically significant (r=0.33, df=14, ns), indicating that, for these fisheries and likely for others, catch data are not good proxies for fishing effort. Although we used catch as the only available measure of how fishing pressure may be distributed across the region, developing reliable estimates of fishing effort across the Mediterranean is a key research priority.

Below we describe the additional data layers used in these analyses.

Invasive species

Halpern et al. (2008) modeled the incidence of invasive species as a function of the amount of cargo traffic at a port, on the basis of results from other studies showing a relationship between these two variables and in the absence of actual data for the global distribution of invasive species (Fig. S2). In this analysis, we replaced this layer with data on the actual distribution of a subset of invasive species in the Mediterranean (Fig. S3). We used maps compiled by The Mediterranean Science Commission (CIESM Atlas of Exotic Species, www.ciesm.org) on the distribution of exotic crustaceans, fish, molluscs and macroalgae in the Mediterranean. CIESM provided low resolution, per-species images showing the area impacted by each invasive species. From these images we derived spatial maps of species distribution by georeferencing the coordinates contained within the images with our study area, which converts the image coordinates into geographic coordinates via a transformation function. We used the GDAL software (http://trac.osgeo.org/gdal/wiki/FAQGeneral) with seven control points and a second order polynomial fit. These georeferenced maps were then digitized to capture the areas of frequent, infrequent and local occurrence. Finally, these polygons were rasterized to the resolution and extent of the overall model.

Based on a review of the literature (e.g., Boudouresque & Verlaque 2002; Galil 2009; Occhipinti et al. 2010; Zenetos et al. 2010) and consultation with experts (B. Galil, National Institute of Oceanography, Haifa, Israel; and A. Zenetos, Hellenic Centre for Marine Research, Anavissos, Greece), we selected a subset of 20 harmful invasives with documented ecological impacts. This species list comprised 10 macroalgae: *Acrothamnion preissii, Asparagopsis armata, Lophocladia lallemandii, Womersleyella setacea, Sargassum muticum, Stypopodium schimperi, Caulerpa racemosa, Caulerpa taxifolia,Halophila stipulacea, and Codium fragile;*
6 molluscs: *Ceratostoma inornatum, Musculista senhouisia, Rapana venosa, Ruditapes philippinarum, Pinctada radiata, and Strombus persicus*; the crab *Percnon gibbesi*; and 3 fishes: *Siganus luridus, Signaus rivulatus, and Lagocephalus scleratus.*

We overlayed the distributions of each of these species to produce a datalayer of the number of harmful marine invasives reported within each 1 km^2 of intertidal and shallow subtidal (down to 60 m depths) coastal habitat.

Oil spills

Pollution at sea, originating from shipping (e.g., oil spills, waste discards, oils and fuels leakage) was modeled in Halpern et al (2008) based on the data on shipping traffic (see above, and details provided in Halpern et al. 2008) (Fig. S4). Here we replaced this data layer with data on the actual occurrence and magnitude of oil spills within the Mediterranean. We obtained data on the occurrence and magnitude of ship accidents resulting in oil spills between 1977-2009, compiled by the Regional Marine Pollution Emergency Response Centre for Mediterranean Sea (REMPEC) (Fig. S5). Using the included attribute of oil released quantity, we performed the same plume modeling techniques used for our land-based threats (see above, 'land based drivers') to distribute the quantities into the surrounding ocean waters. For those accidents where no release quantity was provided, we used a default value of 1 ton released. A total of 93,600 km^2 was affected by one or more accidents.

Climate change (SST increase)

Halpern et al. (2008) quantified temperature warming as the difference in the frequency of positive temperature anomalies between the first and last 5 years of a twenty-year time series (1985-2005) of sea surface temperature (SST) data from remote sensing (Fig. S6). In the present analysis we replaced this data layer with estimates of rates of change in SST as these represent a more direct measure of temperature warming. The detailed spatial analysis of rates of change in SST, utilizing remote sensing data for 1985-2006, is taken from Nykjaer (2009). As shown in Nykjaer (2009) the change in temperature is not constant throughout the year but occur primarily

during summer months. In order to capture the seasonal influence of SST we repeated all analyses using either the annual or the summer (calculated for the months of June, July and August) SST rate of change. However, the resulting cumulative impact maps using either the mean annual or summer SST rate of change were very similar. The map of the resulting annual SST anomalies for the entire area and selected sub areas is shown in figure S7.

Risk of hypoxia

The semi-enclosed setting of the Mediterranean and the high coastal population density make coastal areas of the Mediterranean Sea prone to anthropogenic eutrophication and associated formation of hypoxic and anoxic waters. To capture this pressure on Mediterranean coastal marine ecosystems, we included in our analysis an additional data layers representing the risk of development of hypoxic conditions. The risk of development of low-oxygen conditions was predicted based on a biophysical model (OxyRisk) developed by Djavidnia et al. (2005). We used the modeled output to identify areas susceptible to hypoxia (Fig. S8).

Coastal erosion, aggradation, and armoring.

Data on coastal erosion, aggradation (Fig. S9), and armoring (Fig. S10) were available, only for the Mediterranean countries that belong to the EU, from the EU project CORINE managed by the European Environmental Agency. This program was set up with the aim to gather and integrate information on the state of the environment in the European Community, as a basic instrument for the European Environmental policy. The concrete goal of a component of this project, CORINE – coastal erosion (CCEr), was to provide a description of European coastal systems' morphology and highlight the causes of its modification. Such causes fall under two nonexclusive categories: (i) major natural events (subsidence and rising of the marine level; storms and marine overhigh tides, seisms, mass movements); (ii) human actions causing movements of sediments (reduction of river contributions; development of estuaries, engineering of the coast; construction on the

coastal dunes; harbor work and coastal defense construction; sediment, water, gas or oil extraction).

Data layers are downloadable free of charge from the EEA web site:
http://dataservice.eea.eu.int/dataservice/metadetails.asp?table=COASTEROS&i=1
The data is provided as a series of line segments of the EU coastline, and includes attributes on each segment indicating the level of erosion, aggradation and coastal defenses present within that line segment. For our purposes, we classified the erosion and aggradation classes based on the certainties included in the attribute data, with confirmed reports of erosion and aggradation taking priority over undocumented reports. These classes were then used to estimate the extent and impact of these drivers, which resulted in: 33,807 km^2 aggrading, 80,259 km^2 eroding, and 71950 km^2 undergoing defense.
Datalayers on the presence of harbors or coastal defense structures, and on the erosion or aggradation of the coastline are delivered as vector format with a scale 1:100,000.

Urbanization trends

Coastal urbanization trends (Fig. S11) data were also produced by the EU project CORINE. This datalayer was downloaded from http://www.eea.europa.eu/data-and-maps/data/urban-morphological-zones-changes-1990-2000-umz1990-2000-f1v0-1
The steps involved in generating this datalayer started with the definition of an Urban Morphological Zone (UMZ) as “a set of urban areas laying less than 200m apart”. These urban areas were defined following the Corine Land Cover Core classes:

111 – Continuous urban fabric

112 – Discontinuous urban fabric

121 – Industrial or commercial units

141 – Green urban areas

Enlarged core classes: 123 (Port areas), 124 (Airports) and 142 (Sport and leisure facilities), are also considered if they are neighbors to the core classes or to one of them touching the core classes.

122 (Road and rail networks) and 511 (Water courses), when neighbors to the enlarged core classes, cut by 300m buffer.

Forests & scrub (311,312,313,322,323,324), when they are completely within the core classes.

An UMZ layer was obtained, which was rasterized (100 m resolution). UMZ Changes were calculated by comparing data from 1990 with 2000 and looking for those polygons existing only in one year (i.e. 1990 or 2000). Most changes were positive changes, indicative of urban sprawl (i.e. new UMZ areas between 1990 and 2000), while negative changes describe the reduction of a certain UMZ between 1990 and 2000.

Normalization of driver data layers

Nearly all of the anthropogenic driver data had extreme left-skewed distributions with very small numbers of extremely high outlier data. The extreme outlier data may or may not be precisely accurate, and so we sought to minimize the impact of this potential source of error on our model results. To do so we log[X+1]-transformed each data layer, except *benthic structures*. *Benthic structures* were treated as binary data since presence of an oil rig is an all-or-nothing event. All data layers were then rescaled between 0-1, with the highest log-transformed value for each driver set = 1. The transformation of data appropriately reduces the effect of extreme outliers when rescaling the data to assign the relative impact of different levels of the anthropogenic drivers considered here (Malczewski 2000), while rescaling allows for direct comparison among drivers with dramatically different native scales and units of impact. We also rescaled all driver layers without log-transformation of the data for comparison. This method preserves the true relative magnitude of the data; we found no qualitative differences in our results (not shown) and

so focus on those that derive from log-transformed data. Our approach assumed a linear relationship between driver magnitude and impact on ecosystems. This assumption ignores thresholds that likely exist but are known for very few driver-by-ecosystem combinations, but it allows for direct comparison between and among drivers.

Data representation and projection

To create a uniform coastline (land-sea interface) against which we compared all driver and ecosystem data, we used the SRTM30-PLUS data (http://topex.ucsd.edu/WWW_html/srtm30_plus.html) that represents merged SRTM30 and ETOPO2 data. Land-based data that occurred within the ocean or ocean-based data that occurred on land, in reference to this data mask, were clipped and removed. This issue emerges solely for coastal cells, where a 1km^2 region has both land and ocean in it but must be assigned a binary value (land or ocean) or where the two layers do not meet (i.e. a gap exists). Our approach of applying a uniform land mask ensured consistency across all data used in our analyses, but removed or added area from coastal marine ecosystems. Shallow soft bottom and rocky reef ecosystems gained area as a result of this process due to the need to fill gaps between land and ocean datalayers – we expanded neighboring ocean substrate data into these gaps. In contrast, seagrass ecosystems lost area since these habitats are in very shallow water and so were more prone to being classified as land.

All data were represented at 1km^2 resolution, even though several layers had native resolutions at coarser scales. In doing so, we assumed the coarse-scale value was evenly distributed across all 1km^2 cells within that region. For the climate change drivers (UV anomalies, SST change, and changes in ocean acidification), this assumption is reasonable given the scale at which those drivers act. The land-based drivers, human population data, and oil/gas development data were all at our native 1 km^2 resolution, and spread of these data into the ocean at the same resolution is reasonable. Regardless, when coarse-scale data are distributed equally to

all 1 km^2 cells within the larger area, the coarser scale pattern is essentially recreated while the finer resolution information is preserved where and when it is appropriate. Finally, prior to all analyses, we converted all data to the WGS84 Mollweide projection as it is an accurate single global projection that preserves area and allows data transfer and analysis among operating systems and software.

REFERENCES

Boudouresque CF, Verlaque M (2002) Biological pollution in the Mediterranean Sea: invasive versus introduced macrophytes. Mar. Poll. Bull. 44: 32–38.

Djavidnia S, et al. (2005) Oxygen Depletion Risk Indices - OXYRISK & PSA V2.0: New developments, structure and software content. European Commission, EUR 21509 EN.

Galil BS (2009) Taking stock: inventory of alien species in the Mediterranean Sea. Biol. Invasions 11(2): 359-372.

Halpern BS, et al. (2007) Evaluating and ranking the vulnerability of marine ecosystems to anthropogenic threats. Conserv. Biol. 21: 1301-1315.

Halpern BS, et al. (2008) A global map of human impact on marine ecosystems. Science 319: 948-952.

Halpern BS, et al. (2009) Mapping cumulative human impacts to California Current marine ecosystems. Conservation Letters 2: 138-148.

Malczewski J (2000) On the use of weighted linear combination method in GIS: common and best practice approaches. Transactions in GIS 4: 5-22.

Nykjaer L (2009) Mediterranean Sea surface warming 1985–2006. Climate Research 39: 11-17.

Occhipinti-Ambrogi A, et al. (2010) Alien species along the Italian coasts: an overview. Biol. Invasions 13: 215-237.

Selkoe KA, et al. (2009) A map of cumulative impacts to a “pristine” coral reef ecosystem, the Papahanaumokuakea Marine National Monument. Coral Reefs 28: 635-650.

Zenetos A, et al. (2010) Alien species in the Mediterranean Sea by 2010. A contribution to the application of European Union’s Marine Strategy Framework Directive (MSFD).
Part I. Spatial distribution. Mediterranean Marine Science 11/2: 381-493.

Table S1. List and characteristics of spatial datalayers representing drivers of change of marine ecosystems used to assess and map cumulative human impacts to the Mediterranean and Black Seas. Data used were obtained from the previous global analysis conducted by Halpern et al. (2008), The Mediterranean Science commission (CIESM), the Regional Marine Pollution Emergency Response Centre for Mediterranean Sea (REMPEC), and the European Environmental Agency (EEA). Eighteen data layers came from Halpern et al.'s global analysis and had Mediterranean wide coverage (Global) while the remaining four were available only for the territorial waters of Mediterranean countries that are part of the EU (EU). *Actual cell size is ~750 m to a side to preserve the relationship with the global data from Halpern et al. (2008), which was 1km centered at the equator vs. a center at 38 degrees north in the present analysis. **Digitized from low resolution images to polygons, then converted to 1km raster.

**Drivers**	**Extent**	**Resolution**	**Source**	**Driver category**
Artisanal fishing	Global	1km*	Halpern et al. 2008	fishing
Benthic structures (oil rigs)	Global	1km	Halpern et al. 2008	sea based
Coastal aggradation (coastal renourishment)	EU	1km	EEA	land based
Coastal engineering (coastal defense and harbors)	EU	1km	EEA	land based
Coastal erosion	EU	1km	EEA	land based

Coastal population density	Global	1km	Halpern et al. 2008	land based
Commercial shipping	Global	lines	Halpern et al. 2008	sea based
Fishing (demersal, destructive)	Global	1.0 degree	Halpern et al. 2008	fishing
Fishing (demersal, non-destructive, high bycatch)	Global	0.5 degree	Halpern et al. 2008	fishing
Fishing (demersal, non-destructive, low bycatch)	Global	0.5 degree	Halpern et al. 2008	fishing
Fishing (pelagic, high by-catch)	Global	0.5 degree	Halpern et al. 2008	fishing
Fishing (pelagic, low by-catch)	Global	0.5 degree	Halpern et al. 2008	fishing
Invasive species	Global	polygons**	CIESM	sea based
Nutrient input (fertilizers)	Global	1km	Halpern et al. 2008	land based
Ocean acidification	Global	1.0 degree	Halpern et al. 2008	climate
Oil spills	Global	1km	REMPEC	sea based
Organic pollution (pesticides)	Global	1km	Halpern et al. 2008	land based

Risk of hypoxia	Global	1.5km	EEA	land based
Sea Surface Temperature change	Global	4km	EEA	climate
Urban runoff (nonpoint inorganic pollution)	Global	1km	Halpern et al. 2008	land based
Urbanization trends	EU	1km	EEA	land based
UV radiation	Global	1km	Halpern et al. 2008	climate

Table S2. Correspondence between descriptors of Good Environmental Status (http://ec.europa.eu/environment/water/marine/ges.htm) and the data layers used in this analysis.

**GES Descriptor**	**Datalayers used in this analysis**
1. Biodiversity	Habitat data layers
2. Non-indigenous species	Invasive species
3. Populations of commercial fish species	Fishing (5 data layers, see Table S1)
4. Food web integrity	Fishing (5 data layers, see Table S1)
5. Eutrophication	Nutrient input (fertilizers), risk of hypoxia
6. Sea floor integrity	Demersal destructive fishing
7. Alteration of hydrographical conditions	Coastal engineering, coastal erosion and aggradation
8. Concentrations of contaminants	Organic pollution (pesticides), urban runoff (nonpoint inorganic pollution), oil spills
9. Contaminants in seafood	Organic pollution (pesticides), urban runoff (nonpoint inorganic pollution), oil spills
10. Marine litter	Coastal population density, commercial shipping
11. Introduction of energy (including underwater noise)	Benthic structures (oil rigs), commercial shipping, urbanization trends

Table S3. Average, sum, standard deviation (SD) and maximum values of the cumulative impact score to individual ecosystems (I_E) within the Mediterranean Sea. Depth ranges for different ecosystem types are reported in parentheses.

**Ecosystem**	**avg. I_E**	**sum**	**SD**	**max I_E**
pelagic (<2000m)	1.88	22477210.88	2.34	11.09
deep soft bottom (>2000m)	0.66	7896712.31	1.57	8.33
soft continental slope (200-2000m)	0.52	6168622.41	1.24	7.43
shelf soft bottom (60-200m)	0.22	2580616.91	1.02	9.75
sublittoral soft bottom (0-60m)	0.14	1615186.52	0.64	9.87
deep pelagic (>2000m)	0.12	1435376.81	0.36	5.17
deep hard bottom (>2000m)	0.07	872366.74	0.40	3.60
hard continental slope (200-2000m)	0.06	691979.82	0.40	6.46
shelf hard bottom (60-200m)	0.05	537615.56	0.51	11.90
sublittoral hard bottom (0-60m)	0.04	528421.02	0.46	12.84
intertidal mud flats	0.03	335382.39	0.31	12.99
salt marsh	0.03	316368.69	0.28	12.24
rocky intertidal	0.03	310808.18	0.28	13.30
suspension-feeding reef	0.02	285541.27	0.24	13.89
sandy beach	0.02	216431.11	0.15	7.58
seagrass	0.02	185143.96	0.15	8.28
seamount	0.01	120830.41	0.02	4.57

Table S4. Average, minimum, maximum, standard deviation (SD) and coefficient of variation (CV) of the cumulative impact scores (*Ic*) within territorial waters of Mediterranean and Black Sea EU member states.

Country	avg. Ic	min Ic	max Ic	SD	CV
Slovenia	15.3	3.7	59.4	11.0	71.5
Cyprus	12.7	0.3	62.3	4.0	31.3
Spain	10.7	0.1	87.8	6.2	58.3
Monaco	10.6	6.4	52.4	7.0	65.5
Italy	10.2	0.1	97.4	6.9	67.6
France	10.1	0.0	82.6	7.1	70.5
Greece	9.5	0.0	66.0	5.0	52.9
Malta	9.0	0.5	71.1	5.8	64.7
Bulgaria	7.1	0.8	62.5	4.4	61.8
Romania	6.2	0.4	72.7	2.9	46.4

FIGURE LEGENDS

Figure S1. Correlation between fishing effort (in No. Hooks/year, for the years 1999-2000) and catch (in metric tons/year, normalized to local primary productivity, see Halpern et al. 2008) for pelagic longlines.

Figure S2. Modeled distribution of marine invasive species. This layer was used in the global analysis by Halpern et al. (2008), and was replaced here with a data layer on the actual distribution of 20 harmful invasive species (Fig. S3).

Figure S3. New data layer used in these analyses on the distribution of harmful invasive species in Mediterranean coastal waters.

Figure S4. Modeled pollution at sea layer used in the global analysis by Halpern et al. (2008). This layer was replaced with actual data on the occurrence and magnitude of oil spills in the Mediterranean (Fig. S5).

Figure S5. Occurrence and magnitude of accidents resulting in oil release (data from REMPEC). This data layer was used in the present analyses.

Figure S6. Frequency of positive temperature anomalies used in the global analysis by Halpern et al. (2008). In the present analysis, this data layer was replaced with rates of sea surface temperature increase (Fig. S7).

Figure S7. Annual average rate of increase in SST (between 1985-2006).

Figure S8. Risk of hypoxia.

Figure S9. Coastal erosion (red) and aggradation (green) along the coastlines of Mediterranean EU countries.

Figure S10. Coastal armoring (black) along the coastlines of Mediterranean EU countries.

Figure S11. Urbanization trends within Mediterranean EU countries.

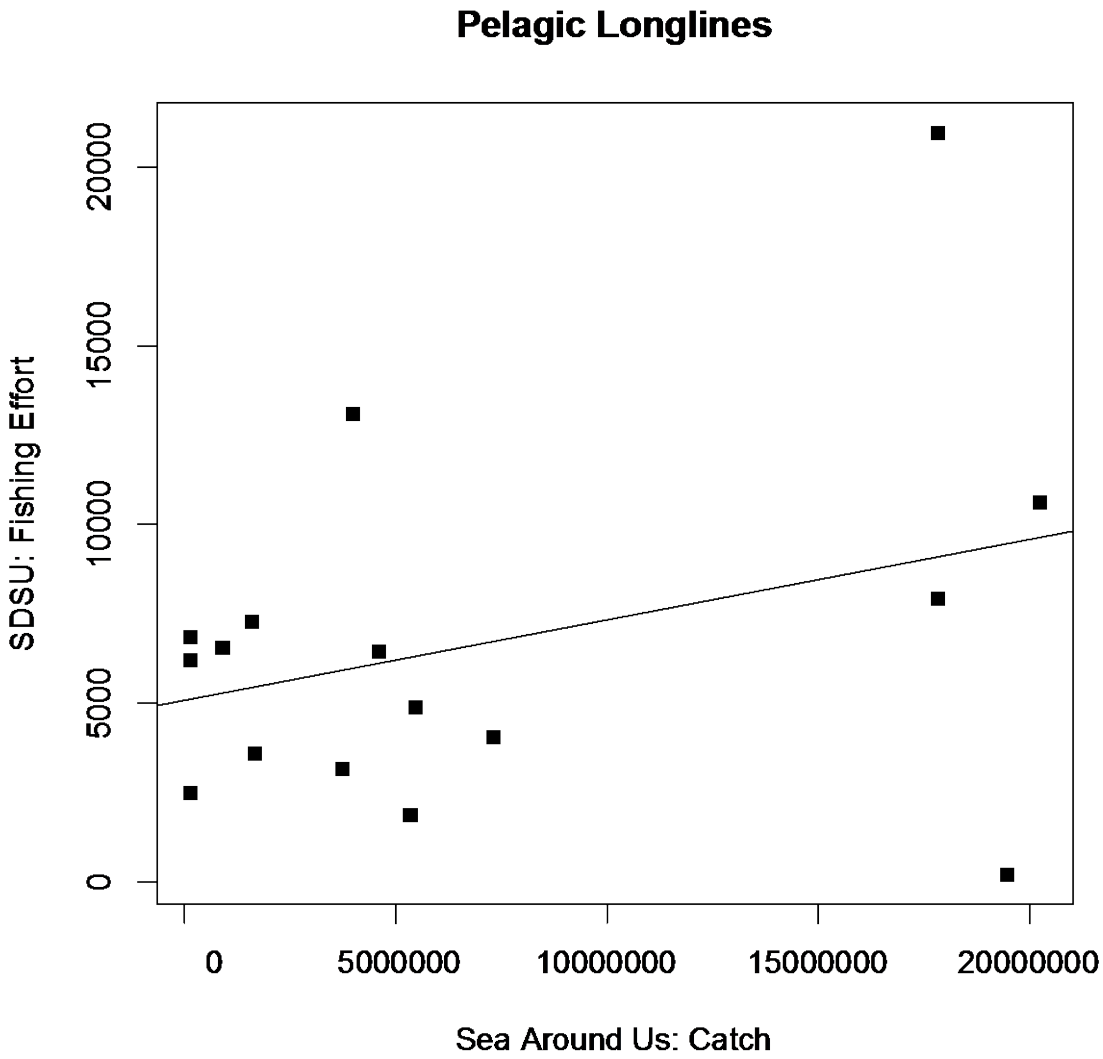
Pelagic Longlines
SDSU: Fishing Effort
0
5000
10000
15000
20000
0
5000000
10000000
15000000
20000000
Sea Around Us: Catch

Figure S1

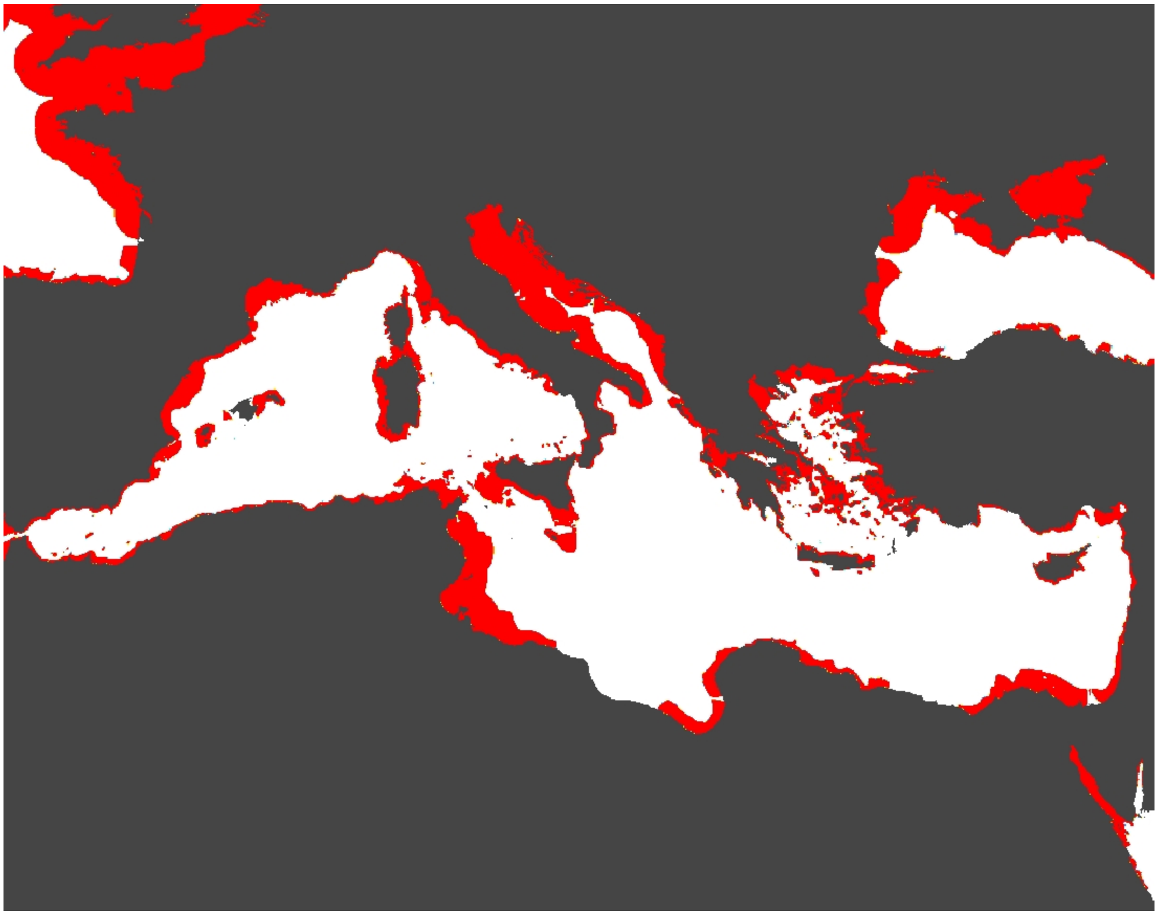

Figure S2

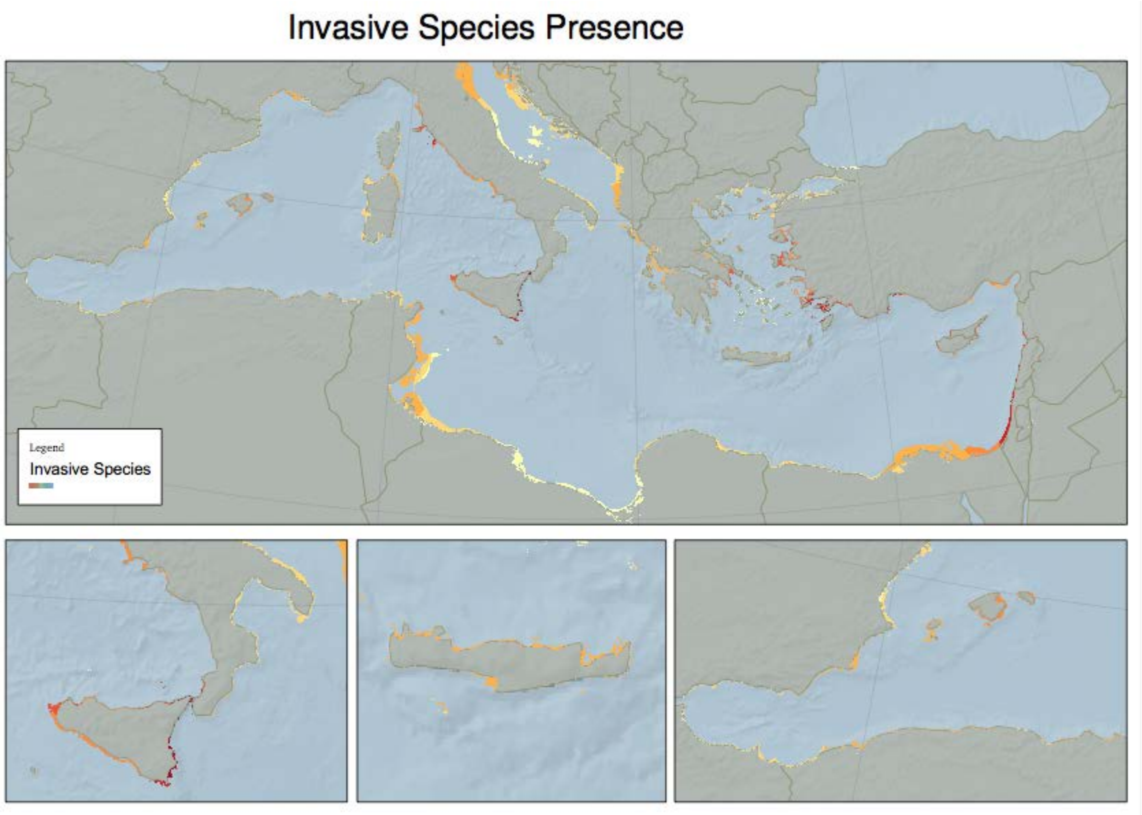

Figure S3

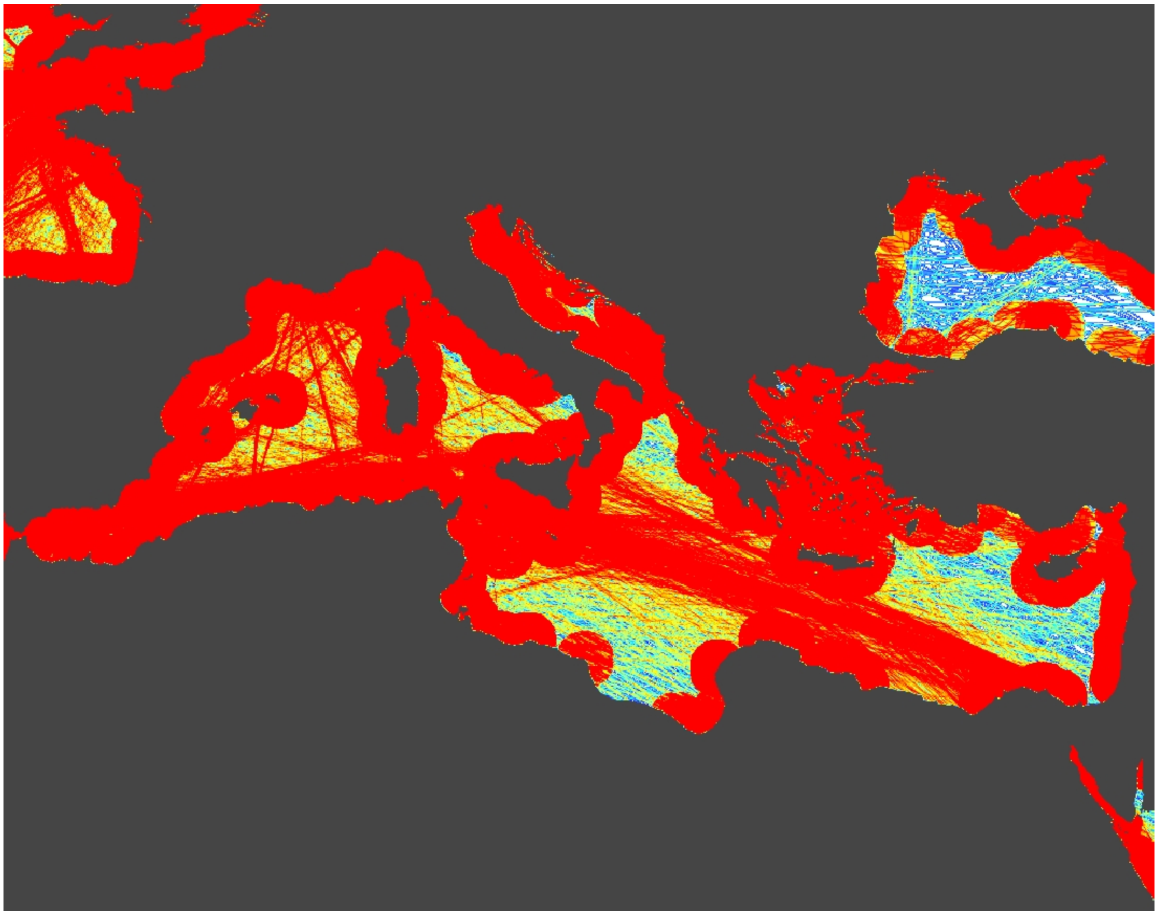

Figure S4

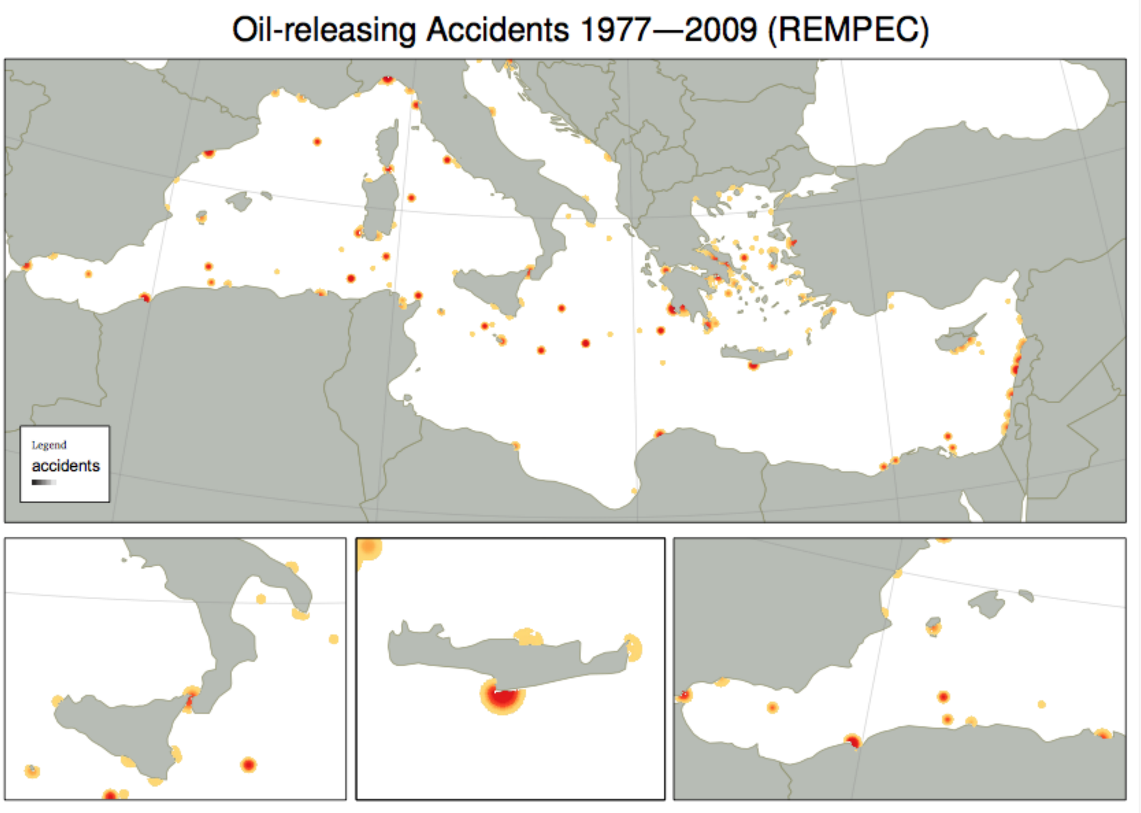
Oil-releasing Accidents 1977—2009 (REMPEC)
Legend
accidents

Figure S5

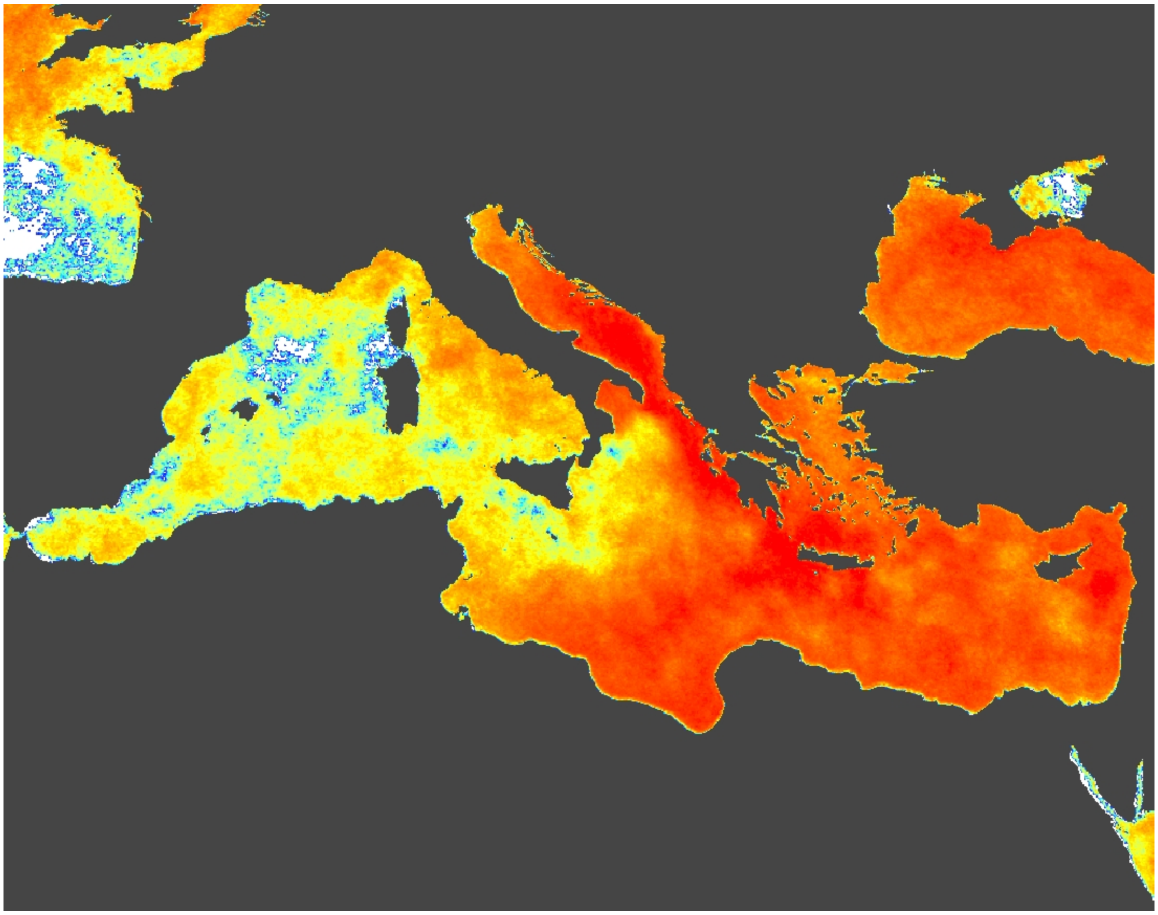

Figure S6

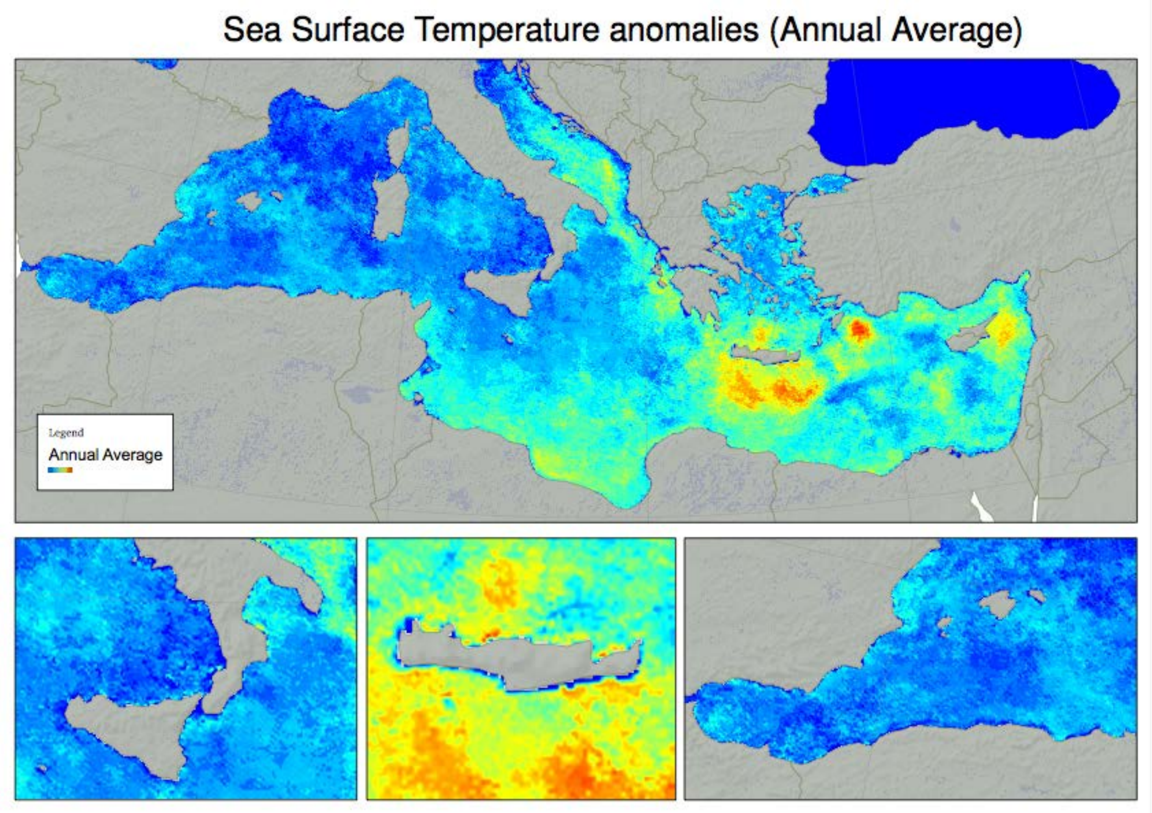
Sea Surface Temperature anomalies (Annual Average)
Legend
Annual Average

Figure S7

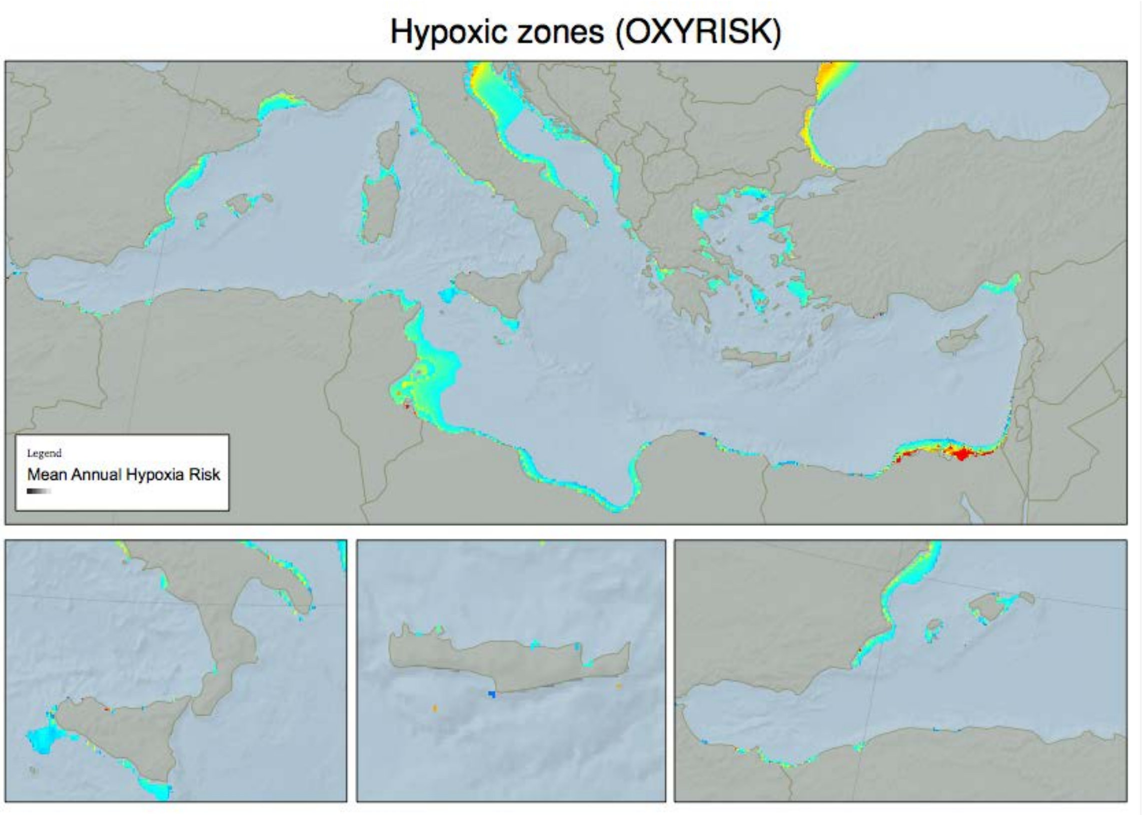

Figure S8

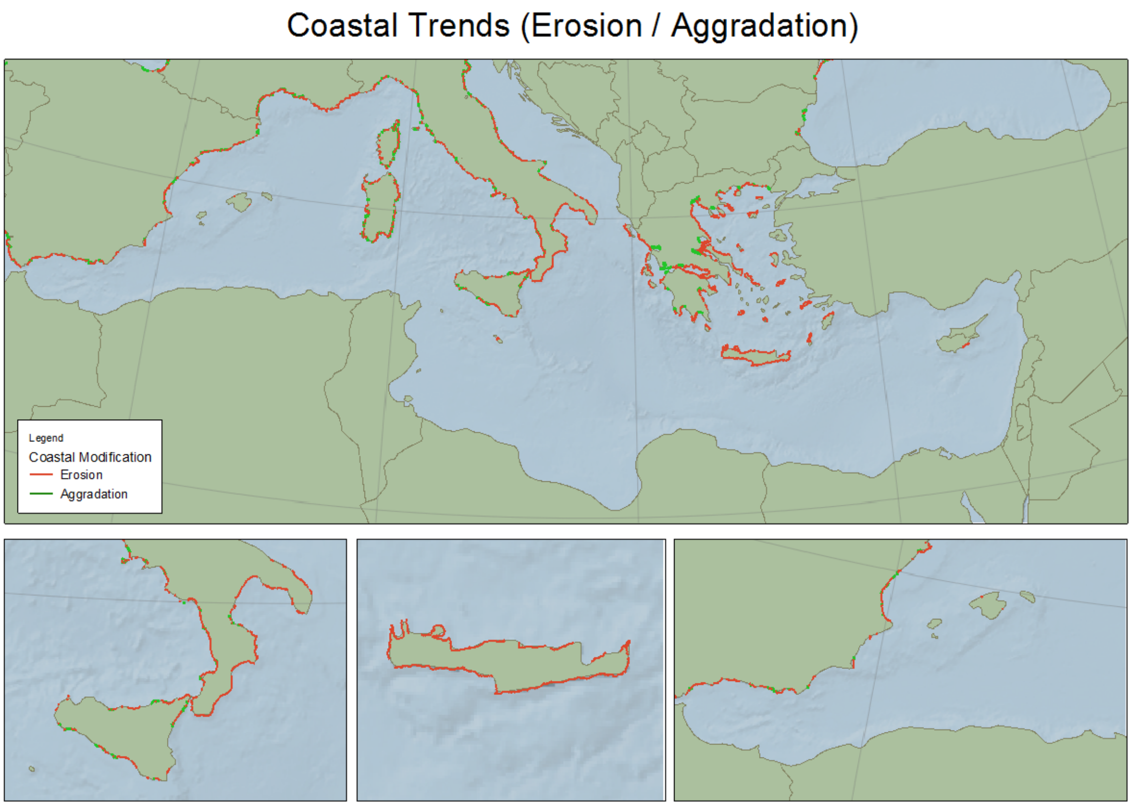
Coastal Trends (Erosion / Aggradation)
Legend
Coastal Modification
Erosion
Aggradation

Figure S9

Coastal Engineering

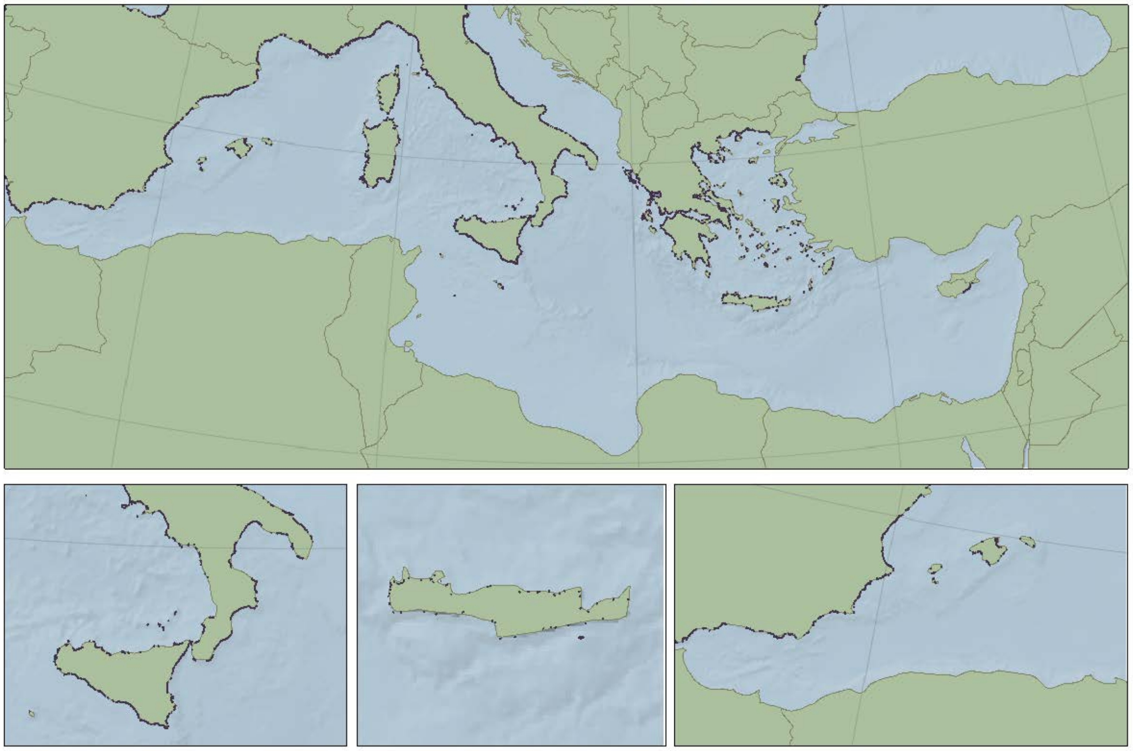

Figure S10

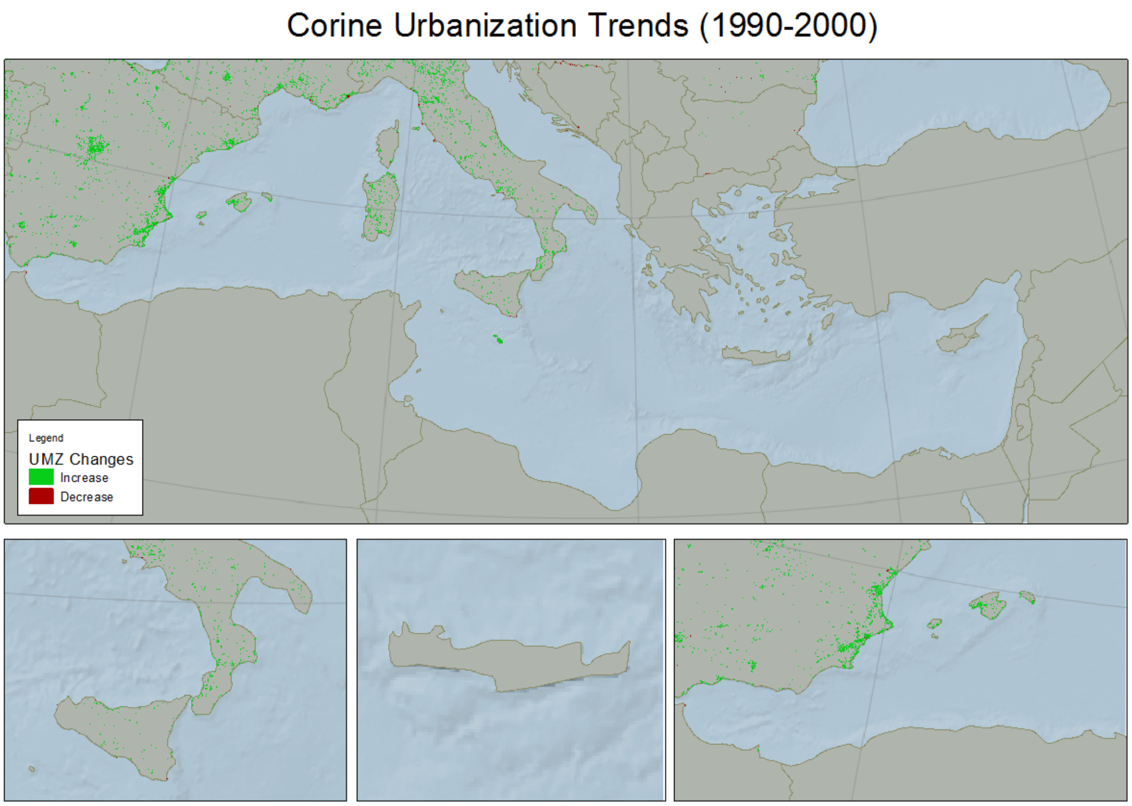
Corine Urbanization Trends (1990-2000)
Legend
UMZ Changes
Increase
Decrease

Figure S11
